# P-26. Clinical Presentation, Management, and Outcome of Aortic Endograft Infections

**DOI:** 10.1093/ofid/ofaf695.255

**Published:** 2026-01-11

**Authors:** Haitham Alaithan, Sarwat Khalil, Muhammad R Sohail

**Affiliations:** Houston Methodist Hospital , Houston, TX; Baylor College of Medicine, Houston, Texas; Baylor College of Medicine, Houston, Texas

## Abstract

**Background:**

Endovascular aneurysm repair involves placement of an endograft within the aorta, either thoracic (TEVAR) or abdominal (EVAR). Aortic endograft infection (AEGI) is rare but with high morbidity and mortality. Management requires a multidisciplinary team approach, but literature is limited. This study describes clinical presentation, management, and outcomes of AEGI at Baylor St. Luke's Medical Center (BSLMC).

**Figure:**
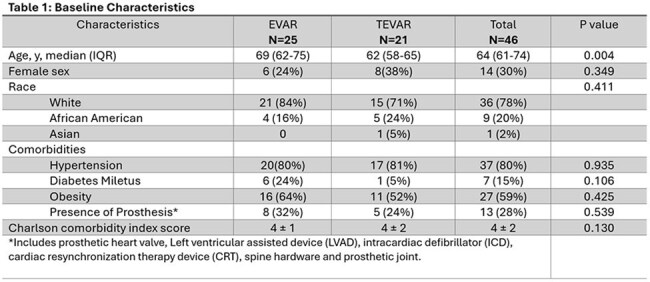

**Figure:**
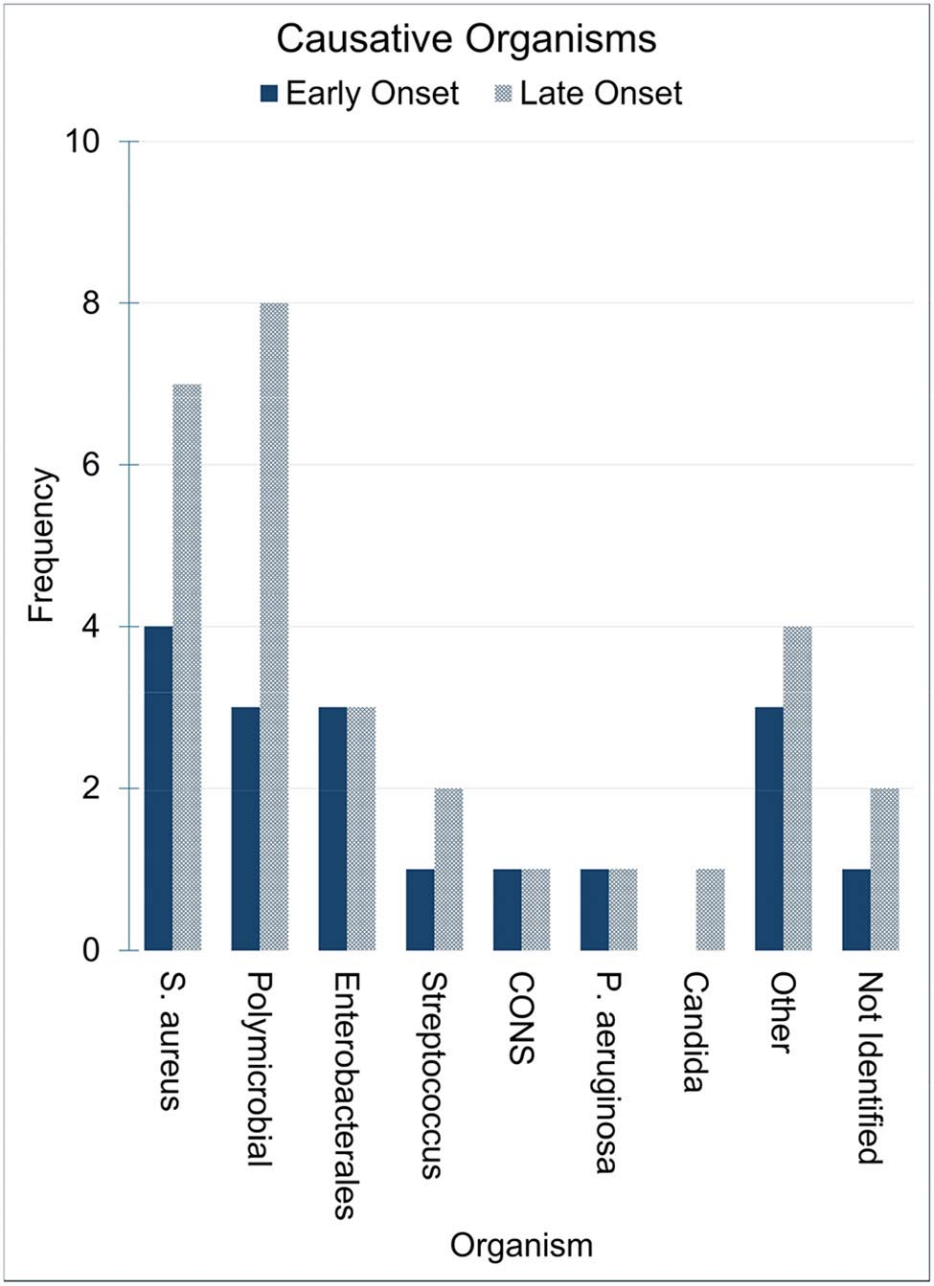

**Methods:**

We conducted a retrospective descriptive review of AEGI at BSLMC between January 2017 to August 2024. Patients were screened using the ICD-10 codes “T82.7, Z95, and Z86.79” and defined using the Management of Aortic Graft Infection Collaboration (MAGIC) criteria.

**Figure:**
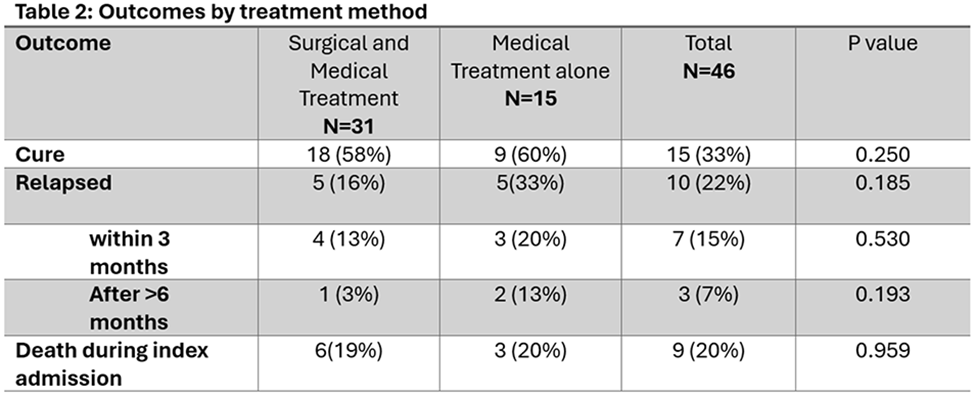

**Results:**

We screened 517 patients and 46 met AEGI criteria. EVAR and TEVAR accounted for 54% and 46%, respectively. The majority (63%) presented with late-onset infections. Common clinical features included fever (54%) and sepsis (48%). Abdominal pain was more prevalent in EVAR compared to TEVAR (56% vs 14%), P=0.004. CT (85%) and PET scan (43%) were primary imaging modalities. S. aureus and polymicrobial infections were each found in 24% of cases. TEVAR patients were more likely to have positive blood cultures compared to EVAR patients, 71% vs. 40%, p=0.033. The majority of patients were treated with a combination of graft surgical resection and antibiotics (67%). β-lactams were the most frequently used antibiotics for treatment (77%). Tetracyclines were the most used for suppression (44%). Cure rates were similar between surgical-medical treatment vs medical treatment alone; 58% vs 60%. Both groups showed a similar mortality rate during the index admission, 19% vs 20%. Medical management alone was associated with higher relapse rates (33% vs. 16%, p=0.185).

**Conclusion:**

In our study, most patients presented with a late-onset infection. S. aureus and polymicrobial infections were most frequent. Although surgical and medical management showed overall similar cure rates compared to medical therapy alone, relapses were more frequent in patients who were treated only medically, though this difference was not statistically significant.

**Disclosures:**

Muhammad R. Sohail, M.D, Angiodynamics: Advisor/Consultant|Angiodynamics: Honoraria|Karius Inc: Advisor/Consultant|Karius Inc: Honoraria|Philips: Advisor/Consultant|Philips: Honoraria

